# Enhancing Physician Resilience to Generative AI: Multilevel Framework for Shared Authority, Verification, and Skill Preservation

**DOI:** 10.2196/88058

**Published:** 2026-06-24

**Authors:** Hongxia Pan, Jialin Liu, Siru Liu

**Affiliations:** 1 Rehabilitation Medicine Center and Institute of Rehabilitation Medicine, West China Hospital of Sichuan University, Chengdu, Sichuan, China; 2 Department of Medical Informatics, West China Hospital of Sichuan University, Guoxue Xiang Street, Chengdu, Sichuan, 610041, China, 86 28-85422416; 3 Department of Otolaryngology-Head and Neck Surgery, West China Hospital of Sichuan University, Chengdu, Sichuan, China; 4 Department of Biomedical Informatics, Vanderbilt University Medical Center, Nashville, TN, United States

**Keywords:** cognitive load, authority, accountability, artificial intelligence, AI, generative artificial intelligence, generative AI, clinical safety, physician

## Abstract

As generative artificial intelligence (AI), particularly large language model–based tools, is increasingly integrated into diagnosis, triage, decision support, and treatment planning, it offers potential gains in efficiency and information access. However, real-world deployment also introduces important risks, including hallucinations, miscalibrated confidence, automation bias, and increased verification burden on physicians. This burden may divert attention from independent clinical reasoning, contribute to deskilling, and increase vulnerability when models fail silently or perform poorly in unfamiliar clinical contexts. Existing AI governance frameworks emphasize data quality, transparency, accountability, and ethical deployment, but pay less attention to physician-facing resilience, defined in this paper as the capacity to sustain independent and safe clinical judgment when collaborating with generative AI. In this viewpoint, we propose a multilevel governance framework organized around 3 coordinated domains: cognitive workload shaping, clinical authority governance and allocation, and organizational safety governance and accountability. Together, these domains aim to reduce verification burden, preserve physician decisional authority, and align institutional oversight with safe and context-sensitive AI use. The framework includes mechanisms such as risk-sensitive verification triggers, bounded delegation, structured interprofessional review, and organizational monitoring to support safe clinical integration while minimizing avoidable workflow disruption. At the same time, implementation may be limited by workflow friction, alert fatigue, variable institutional resources, and the need for ongoing monitoring and recalibration to ensure that safeguards remain clinically useful rather than burdensome. Accordingly, this paper outlines a structured governance framework to guide safer integration of generative AI into clinical care and inform future evaluation across specialties, workflows, and institutional settings.

## Introduction

Large language model–based generative artificial intelligence (AI) is rapidly extending from documentation support to core clinical activities, including diagnosis, clinical decision support (CDS), triage, treatment recommendation, and preventive care planning [1-6]. Early evaluations suggest potential gains in timeliness and efficiency; however, deployment in real-world clinical settings introduces sociotechnical risks with direct implications for safety and professional competence [7,8]. Generative AI can produce fluent but incorrect outputs (hallucinations); express confidence that is not supported by the underlying evidence; and perform inconsistently under distribution shift, prompt artifacts, or algorithmic bias, potentially leading to clinically unjustified differences across demographic or socially marginalized groups [9]. In this paper, “distribution shift” refers to reduced performance when the AI encounters patients, settings, or data patterns that differ from its training data, and “prompt artifacts” refers to misleading changes in output caused by superficial wording or formatting differences rather than clinically meaningful distinctions. These risks impose a substantial verification burden: physicians must divert time and attention from intrinsic clinical reasoning to source checking, uncertainty appraisal, and contextual fit assessment, thereby increasing extraneous cognitive load [10-12]. In addition, assigning complex integrative tasks to AI may contribute to deskilling as independent diagnostic synthesis and risk communication decline, leaving physicians more vulnerable during model failures or out-of-distribution conditions [13,14]. Coupled with automation bias and overtrust, these pressures create structural vulnerabilities that cannot be mitigated solely through traditional safety training or single-discipline governance [15].

We use “resilience” in the sense established in resilience engineering: the capacity of a system or its actors to sustain safe and effective functioning under variable, degraded, or unanticipated conditions [16,17]. This usage differs from narrower psychological accounts that emphasize individual emotional coping or recovery from adversity. Applied to the physician-AI interface, physician resilience refers to the sustained capacity for independent clinical judgment when collaborating with systems whose outputs may be uncertain, miscalibrated, or context inappropriate. Although AI governance research has extensively examined data quality, transparency, and ethical principles, less attention has been paid to the combined cognitive, professional, and organizational conditions needed to preserve this resilience in physician-AI collaboration.

Sustaining this resilience depends on preserving physician autonomy. Autonomy in this case does not mean isolated clinical discretion or resistance to AI assistance. Rather, it refers to the physician’s professionally accountable capacity to interrogate, contextualize, accept, modify, or override AI-generated suggestions based on clinical evidence, patient values, uncertainty, and situational constraints [18,19]. The relationship between resilience and autonomy is reciprocal: resilience requires preserved autonomy so that physicians can adapt safely when AI outputs are incomplete, biased, or miscalibrated; autonomy, in turn, requires resilient cognitive, professional, and organizational conditions that prevent clinical judgment from being displaced by automation bias, excessive verification burden, or institutional pressure to defer to algorithmic outputs [7,10,11,15]. Therefore, the governance challenge is not only whether generative AI outputs are accurate but also whether clinical environments preserve physicians’ capacity to remain accountable decision-makers within a model of shared human-AI authority.

**Figure 1.**
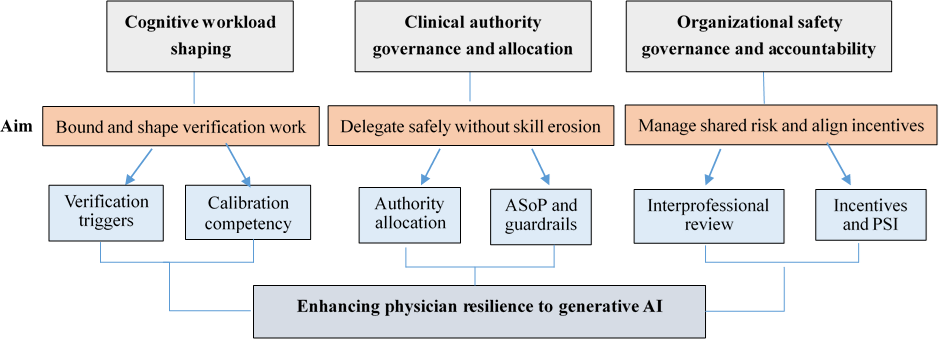
Three-pillar governance model for enhancing physician resilience to generative artificial intelligence (AI). ASoP: automation scope of practice; PSI: protected safety investment.

To address this gap, we propose a multilevel governance framework organized around 3 coordinated domains (Figure 1): cognitive workload shaping, clinical authority governance and allocation, and organizational safety governance and accountability. This resilience-centered framing explains why governance must address not only model performance but also the cognitive, professional, and institutional conditions under which physicians can sustain safe judgment and accountable authority in AI-supported care. Together, these domains aim to bound and rebalance verification work, preserve physician decisional authority while enabling safe delegation, and strengthen institutional accountability for shared risk. Figure 1 depicts the relationships among these domains and their associated governance mechanisms. To clarify the 2 implementation levels of the framework, “institutional recommendations” refers to governance design, resource allocation, workflow integration, monitoring, and oversight structures. In contrast, “physician-facing recommendations” refers to verification practices, uncertainty appraisal, patient communication, sign-off, and preservation of clinical judgment. These levels cut across all 3 domains rather than mapping onto separate domains because safe physician action often depends on institutional design, resources, and oversight.

This viewpoint presents a normative, concept-driven framework developed through interdisciplinary, literature-informed synthesis rather than formal empirical data collection or expert consensus procedures. We drew on 5 research areas: resilience engineering, clinical reasoning and metacognition, CDS stewardship, AI governance and regulation, and emerging evidence on automation bias and AI-induced deskilling. From these, we identified recurrent risks at the physician-AI interface and organized corresponding governance responses across the 3 domains. The framework is intended to guide implementation and evaluation of physician-facing safeguards for generative AI in clinical care and extend rather than replicate governance approaches developed for traditional rule-based CDS given the distinct properties of generative AI outputs.

## Cognitive Workload Shaping

### Overview

In a busy outpatient clinic, a physician uses an electronic health record–integrated generative AI tool to summarize a patient’s history and suggest possible explanations for subacute dyspnea. The summary is fluent but omits a recent medication change and presents several low-probability diagnoses as a coherent leading narrative. The resulting risks are 2-fold: factual incompleteness or distortion and cognitive bias induced by a plausible but misleading narrative frame. The physician may use the AI-framed explanation as an anchor, divert attention to verifying low-probability claims, or overlook clinically important information omitted from the summary. Therefore, the central governance challenge is to reduce verification burden while preserving independent clinical reasoning when AI outputs are coherent but epistemically unreliable.

### Verification Triggers and Proportionate Safeguards

Institutions should classify verification triggers using a structured matrix based on 4 dimensions: (1) clinical harm severity if the AI-supported recommendation is wrong; (2) irreversibility and time criticality of the downstream action; (3) uncertainty or conflict intensity, including model uncertainty, weak provenance, or disagreement between AI output and clinician assessment; and (4) equity sensitivity, referring to decisions in which algorithmic bias may plausibly distort recommendations [20,21]. Because algorithmic bias is difficult for individual physicians to detect case by case, equity sensitivity should be operationalized primarily as a system-level trigger supplemented by clinician judgment [20,22]. Institutions should predefine equity-sensitive contexts using local validation results, known subgroup performance gaps, and high-impact domains that affect access, triage, diagnosis, treatment intensity, or follow-up [23,24]. The trigger may activate automatically when an AI-supported recommendation involves these contexts, when patient characteristics fall outside the model’s validated population, or when local audits identify subgroup-specific performance concerns [20,25]. Demographic variables used for this purpose should support bias surveillance rather than justify differential clinical treatment. Clinicians should also be able to invoke the trigger when they identify contextual vulnerability or discordance between the AI output and the patient’s clinical or social context.

Lower-tier triggers involve reversible, low-uncertainty tasks and require brief verification, whereas higher-tier triggers should prompt independent reasoning and explicit attestation. Escalation or second review should be reserved for cases involving high severity, irreversibility, time criticality, substantial uncertainty, conflict with clinician judgment, or equity-sensitive contexts. Thresholds may vary by service line, but classification logic should remain explicit, auditable, and periodically recalibrated. Attestation interfaces are designed to discourage verbatim or formulaic rationales, with free-text fields structured to elicit clinical specificity rather than generic confirmation. Mandatory pauses and other high-interruption mechanisms are reserved for high-risk decisions, whereas lower-risk situations rely on passive or minimally disruptive cues. Routine safety governance includes monitoring of override rates, bypass patterns, and clinician-reported burden to ensure that safeguards remain salient rather than becoming background noise [25].

### Uncertainty and Trust Calibration Competency

Institutions should ensure that physician training shifts from basic AI literacy to verification-oriented competencies and calibrated trust, defined as the ability to adjust reliance on AI according to clinical context, risk level, and uncertainty signals [26,27]. Core competencies include interpreting model uncertainty and calibration indicators as reliability signals rather than proxies for ground truth; calibration indicators refer to signals about whether the model’s expressed confidence or uncertainty corresponds to observed correctness in the relevant clinical context [28]. Protocolized plausibility checks against established physiology, guideline constraints, and patient-specific factors are needed, with documented attestation when a check is triggered. Training should also cover prompt hygiene, defined as structuring prompts to reduce ambiguity and prevent misleading context from shaping the model’s response, and contextual integrity, defined as maintaining appropriate boundaries around what clinical information should or should not be included in an AI interaction [29]. These practices require institutional templates and interface guardrails rather than individual technical improvisation. Physicians should also appraise the provenance and recency of model-cited evidence to determine whether AI-synthesized rationales should be accepted, discounted, or escalated [30]. Together, these competencies support calibrated trust and help contain verification burden in AI-supported care.

## Clinical Authority Governance and Allocation

### Overview

In an oncology service, a generative AI system drafts a chemotherapy dosing recommendation for a patient with declining renal function and evolving laboratory abnormalities. The output is plausible but may not reflect local protocols, recent dose adjustments, or temporal trends in laboratory values. Therefore, the central governance challenge is not only whether the recommendation is clinically correct but also who is authorized to decide, verify, and sign off on a high-risk intervention.

### Decisional Responsibility and Role Allocation

In AI-supported clinical care, safe practice depends on clearly distinguishing the AI system’s informational support role from the physician’s decisional responsibility for clinical interpretation, patient communication, and sign-off [22,24]. AI outputs do not carry independent clinical authority and cannot, by themselves, commit a patient to action. Physicians retain responsibility for interpreting outputs; accepting, modifying, or rejecting recommendations; completing required disclosure and consent processes; and signing off on clinically consequential decisions [24,31]. Accountability should be distributed in proportion to operational control: physicians remain responsible for case-specific clinical judgment; institutions remain responsible for local validation, workflow integration, and performance monitoring; and developers remain responsible for model design, governance update, and management of known limitations [22,32]. This allocation preserves physician judgment while avoiding the inappropriate concentration of liability on physicians for system-level AI failures. In value-sensitive or preference-dependent decisions, decisional authority ultimately rests with the patient, whereas physicians remain responsible for transparent communication and informed, preference-sensitive decision-making.

At the institutional level, a designated AI governance committee or equivalent multidisciplinary body should approve AI deployment within specific workflows, define local validation requirements, and oversee monitoring and escalation pathways. Such a body should include representation from clinical leadership, informatics, quality and safety, legal or compliance, and relevant specialty expertise. Patient-facing transparency, including disclosure of AI involvement and relevant uncertainty, is a core component of safe human oversight and should be tailored to risk level and use case [22,33]. To make disclosure feasible in time-limited encounters, institutions can provide standardized plain-language phrasing, electronic health record–integrated documentation prompts, and risk-stratified consent workflows [31]. For low-risk uses such as AI-assisted summarization or documentation support, brief disclosure that AI was used and reviewed by the clinician may be sufficient. Clinically consequential recommendations, including AI-supported diagnostic interpretation, medication adjustment, triage prioritization, or treatment planning, warrant communication of 4 core points: (1) AI supported the analysis, (2) the physician reviewed and contextualized the output, (3) uncertainty or alternative interpretations remain, and (4) the patient may ask questions or decline AI involvement where legally and ethically applicable. For example, phrasing such as “An AI-supported tool helped check part of the information, but I reviewed it in light of your clinical situation” can be integrated into consent, shared decision-making, or postvisit documentation workflows. When AI outputs conflict with clinical guidelines, documented patient goals, or physician judgment, a verification trigger should activate predefined escalation pathways, with the resulting review, attestation, and rationale recorded in the audit log [20,26,30].

### Automation Scope of Practice and Safe Delegation

To operationalize safe delegation, institutions should apply automation scope of practice (ASoP) through a stepwise decision process that links task classification, verification triggers, and authority allocation [23,24,32]. First, the workflow is divided into discrete task units, such as summarization, differential diagnosis generation, medication review, triage prioritization, or treatment recommendation. Second, each task is assigned an initial ASoP tier based on clinical risk, verifiability, time criticality, and dependence on contextual physician judgment. Third, verification triggers, including harm severity, irreversibility and time criticality, uncertainty or conflict intensity, and equity sensitivity, are applied to determine whether the task requires a higher level of review. Fourth, authority allocation rules determine who may act on the output, who must supervise or attest, and when escalation or second review is required.

Institutions can apply a 4-tier ASoP rubric (Table 1). Tier 0 includes nondelegable, physician-led tasks involving high-stakes diagnostic or therapeutic judgment, weak verifiability, time-critical consequences, or potentially irreversible harm. Assistive tasks such as draft documentation, summarization, or structured information retrieval fall under tier 1, where AI output is low risk, readily verifiable, and not directly action enabling. Tier 2 applies to supervised analytic support, including preliminary differentials, medication conflict flags, or care plan drafting, for which physician review, contextualization, and explicit acceptance are required before clinical action. At tier 3, automation may proceed within predefined institutional rules, audit trails, and escalation criteria for conditionally delegated workflow actions such as low-risk protocol-based triage support, follow-up scheduling, or routine monitoring reminders [23,32].

**Table 1.** Illustrative automation scope of practice (ASoP) tier assignments for representative clinical tasks^a^.

Example task	ASoP tier	Permitted AI^b^ role	Verification and authority requirements
Drafting or formatting a clinical note	Tier 1: assistive	Drafting or structuring text	Clinician reviews for accuracy and omissions before signing; escalates if hallucinated or incorrect content is detected.
Summarizing prior laboratory results, imaging, or medication history	Tier 1: assistive	Extracting and summarizing defined source information	Clinician verifies source accuracy, temporal relevance, and omissions; escalates if data are incomplete, outdated, or outside validated sources.
Generating preliminary differential diagnoses	Tier 2: supervised analytic	Suggesting candidates but not determining diagnosis	Independent clinician reasoning and explicit acceptance, modification, or rejection; escalate for high uncertainty, conflict, or high-risk presentations.
Flagging medication interactions or renal dose concerns	Tier 2: supervised analytic	Flagging concerns or drafting options	Clinician, with pharmacist input where relevant, verifies history, laboratory trends, and contraindications; escalates high-harm or protocol-conflicting cases for second review.
Protocol-based follow-up scheduling or monitoring reminders	Tier 3: conditionally delegated	Recommending or initiating predefined workflow steps	Allowed only within institutional rules, audit trails, and retrospective monitoring; escalate for status change, missing data, or out-of-protocol cases.
Low-risk protocol-based triage support such as stable outpatient referrals	Tier 3: conditionally delegated	Supporting prioritization within bounded protocols	Bounded by predefined rules; higher-risk presentations escalate to clinician confirmation; monitor for red flags or atypical features.
Final diagnosis, chemotherapy dosing, treatment selection, or withholding urgent care	Tier 0: nondelegable	Providing information or options only	Physician-led judgment with explicit attestation; second review for high-stakes or contested cases; no autonomous execution pathway permitted.

a The categories are examples for local adaptation rather than fixed universal rules.

b AI: artificial intelligence.

Tier assignment should be dynamic rather than fixed. Tasks move toward more restrictive tiers when the patient is clinically unstable, input data are incomplete or unreliable, the output falls outside the model’s validated use context, the recommendation affects vulnerable or underserved groups, or the consequences of error are difficult to reverse. These assessments determine what may be automated, what must remain under physician control, and what safeguards are required [22,23].

## Organizational Safety Governance and Accountability

### Overview

In a large health system, a generative AI tool drafts responses to patient portal messages about new symptoms, possible medication adverse effects, and requests for treatment advice. Some drafts are helpful, whereas others are overly reassuring, fail to identify escalation cues, or conflict with local triage protocols. Although individual physicians may detect some errors during review, safe use depends on organizational monitoring thresholds, audit mechanisms, update oversight, and accountability structures. Therefore, the central governance challenge is not only error detection at the point of care but also ensuring that institutional oversight sustains safe physician judgment beyond individual vigilance alone.

### Targeted Interprofessional Safety Review

For prespecified high-risk scenarios such as chemotherapy dose adjustment, physicians should retain final decisional authority while convening focused interprofessional review with relevant colleagues, including pharmacists, nurses, and specialists [24,34,35]. The purpose is not to duplicate individual verification but to support team-based assessment of feasibility, safety, and cross-disciplinary workflow fit [34-36]. Activation should follow verification triggers with team-level implications, and review intensity and timing should be adapted to clinical urgency [24,32]. In time-critical situations, review may involve rapid, role-explicit confirmation. When immediate action is required before review can occur, documented retrospective review should follow. Workflow interfaces should capture role-specific input and physician attestation in the audit log to support traceability and accountability [32,36].

### Physician-Facing Incentives and Protected Safety Investment

Organizational policy should align incentives with safety rather than throughput alone [23,37,38]. Institutions should establish protected safety investment to support nonautomatable functions that strengthen physician decision-making, including time for complex case consultation, targeted interprofessional review training, and selected skill retention activities for high-risk scenarios [38]. AI-related gains can be documented through auditable operational measures such as clinician time returned, reduced after-hours documentation, or avoided downstream workflow failures rather than assumed from adoption alone. Where direct monetization is constrained by billing context, these measures can inform local business cases, and safety investment may need justification as prospective quality infrastructure. Performance monitoring should emphasize auditable safety process measures such as verification trigger adherence, appropriate override or decline rates, attested sign-off, and time to decision in triggered cases [37].

### Skill Retention and Learning-Oriented Error Review

Skill retention activities should be risk stratified and targeted to high-risk or high-delegation workflows. Where feasible, these activities can be integrated into protected simulation time, case-based continuing medical education, credentialing-linked refreshers, or quality improvement and morbidity and mortality review processes rather than assuming that clinicians have unscheduled downtime [13,38]. Institutions should maintain a local learning-oriented, nonpunitive error taxonomy. This taxonomy would classify AI-related safety events by source and mechanism, including interface design problems, human factor contributors, data or workflow mismatches, and model output failures. The resulting signals can guide focused review, targeted training, and interface improvement rather than individual blame [23,39]. To clarify how the 3 framework domains translate into institution-level and physician-facing responsibilities, Table 2 summarizes the major recommendations, primary responsible parties, and practical implications for implementation.

**Table 2.** Framework recommendations by domain and responsible party.

Framework domain and major recommendation	Primary responsible party	Practical implication
Cognitive workload shaping		
Use risk-sensitive verification triggers and proportionate safeguards	Institution led; physician applied	Institutions define thresholds, interface cues, escalation rules, and monitoring processes; physicians apply triggered checks, document rationales, and escalate when needed.
Develop uncertainty and trust calibration competency	Shared institutional and physician responsibility	Institutions provide training, templates, and interface guardrails; physicians interpret uncertainty, assess provenance, perform plausibility checks, and calibrate reliance on AI^a^ outputs.
Clinical authority governance and allocation		
Clarify decisional responsibility and role allocation	Shared institutional and physician responsibility	Institutions define disclosure, consent, accountability, and escalation pathways; physicians interpret AI outputs, communicate AI-supported reasoning and uncertainty, and retain sign-off responsibility for consequential decisions.
Define an automation scope of practice for safe delegation	Institution led; physician applied	Institutions classify tasks by risk, verifiability, competence, and supervision level; physicians use AI only within approved delegation boundaries and apply required verification.
Organizational safety governance and accountability		
Activate targeted interprofessional safety review for high-risk scenarios	Shared institutional and physician responsibility	Institutions define team review triggers and documentation requirements; physicians retain final authority while seeking pharmacist, nursing, specialty, or other input when triggered.
Align incentives with protected safety investment and auditable performance monitoring	Institution led	Institutions protect time and resources for complex case consultation, targeted training, and safety monitoring while avoiding throughput-only measures of AI value.
Support skill retention and learning-oriented error review	Shared institutional and physician responsibility	Institutions use risk-stratified simulation, continuing education, credentialing-linked refreshers, quality improvement processes, and a nonpunitive error taxonomy; physicians participate in targeted reassessment and remediation when needed.

a AI: artificial intelligence.

## Discussion

### Relation to Existing Knowledge

The proposed framework is informed by and extends several established research areas. The verification trigger mechanism draws on cognitive load theory and meta-cognitive training in medical education [12,40]. The authority allocation construct builds on resilience and error management frameworks, whereas the organizational safety pillar is informed by World Health Organization guidance, the European Union AI Act, and emerging institutional initiatives such as the Trustworthy and Responsible AI Network [26,32,41,42]. These frameworks establish essential system-level expectations for transparency, accountability, human oversight, and institutional readiness but do not specify the cognitive and workflow conditions under which physicians can sustain independent judgment while collaborating with generative AI [26,32]. Physician autonomy literature further informs the authority governance component by clarifying why physicians must retain accountable authority to interpret, modify, or override AI-supported recommendations [18,19]. Emerging evidence on automation bias and AI-related deskilling informs the verification, delegation, and skill retention components by identifying mechanisms that may erode independent clinical judgment [7,13-15]. This framework complements these foundations by focusing more explicitly on physician-facing safeguards, including verification triggers, bounded delegation, calibrated interpretation of uncertainty, and skill retention monitoring.

### Distinct Governance Requirements for Generative AI

This framework extends rather than replicates governance approaches used for traditional rule-based CDS. Conventional alert-based CDS is typically narrower in scope and anchored to prespecified logic; accordingly, governance has often focused on alert thresholds, override appropriateness, and workflow burden [24,25]. In contrast, generative AI systems produce context-sensitive natural language outputs that may appear coherent and persuasive while still being false, inaccurate, biased, or incomplete [28]. Their behavior may also vary with prompt design, the quantity and order of information provided, and model updates [29]. Therefore, governance for physician resilience in AI-supported clinical care should extend beyond traditional alert management to include bounded verification triggers, calibrated interpretation of uncertainty, protection of unaided reasoning, and explicit boundaries for AI use within clinician-led decisions [32].

### Limitations and Implementation Considerations

As a normative, conceptual viewpoint, this framework has several limitations. First, it does not yet provide prospective evidence regarding workflow efficiency, clinician acceptance, patient outcomes, or unintended consequences; feasibility and net benefit require pilot-testing and context-specific evaluation [43,44]. Second, safeguards such as verification triggers, diagnostic time-outs, attestation requirements, and targeted interprofessional review may introduce workflow friction, increased administrative burden, alert fatigue, or delays in time-sensitive settings if applied too broadly [25]. Emergency override pathways may also be overused, underused, or applied inconsistently in practice, requiring explicit criteria and review processes to support timely care without undermining accountability. Third, implementation capacity is likely to vary substantially across institutions, particularly in resource-constrained settings lacking sufficient staffing, digital infrastructure, monitoring capacity, or protected implementation time [23,44]. Fourth, sustaining a living ASoP table and longitudinal skill retention activities requires version control, periodic review, and coordination across multiple functions; institutions will need proportionate update cycles, shared templates, and risk-prioritized review processes to keep these mechanisms sustainable.

Accordingly, the framework should be understood as risk stratified and locally adaptable rather than uniformly applicable. In high-acuity contexts, immediate override with deferred review may be necessary to preserve timely care. Thresholds for verification, delegation, and authority sharing will require ongoing recalibration as models, interfaces, and clinical workflows evolve. Future work should identify which components are most feasible, which provide the greatest safety benefit, and how implementation burden can be minimized while preserving physician judgment. For institutions considering adoption, implementation should proceed incrementally, beginning with governance structures and bounded pilot workflows followed by prospective monitoring, recalibration, and staged expansion, with skill retention monitoring and incentive alignment incorporated from the outset rather than after scale-up [13,43,44].

## Conclusions

Physician resilience in AI-supported clinical care depends on more than improved prompting or isolated human oversight requirements. This viewpoint advances a governance approach to generative AI integration that emphasizes bounded verification, preserved physician judgment, and organizational accountability as conditions for safe clinical use. Future work should prospectively evaluate the feasibility, clinical effects, and implementation burden of these mechanisms across specialties, workflows, and institutional settings. Such evaluation should include bounded pilot studies, mixed methods assessments of physician workload and autonomy, safety event monitoring, equity-focused subgroup analyses, and longitudinal assessments of skill retention.

## Abbreviations

AI: artificial intelligence
ASoP: automation scope of practice
CDS: clinical decision support
